# Can It Happen Again? Using Co‐Produced Theatre to Explore the Challenges Faced by Couples Considering Pregnancy After a De Novo Genetic Diagnosis in a Child

**DOI:** 10.1111/hex.70829

**Published:** 2026-08-26

**Authors:** Alison Kay, Anne Goriely, Alexa Gummow, Lisa Hinton, Arti Patel, Marysia Price‐Nowak, Shwetha Ramachandrappa, Jonathon Wells, Francesca Wicks, Minna Jeffery

**Affiliations:** ^1^ MRC Weatherall Institute of Molecular Medicine University of Oxford Oxford Oxfordshire UK; ^2^ Nuffield Department of Primary Care Health Sciences University of Oxford Oxford Oxfordshire UK; ^3^ Centre for Personalised Medicine University of Oxford Oxford Oxfordshire UK; ^4^ St. Anne's College, University of Oxford Oxford Oxfordshire UK; ^5^ ‘Making it Personal’ Co‐production Panel University of Oxford Oxford Oxfordshire UK; ^6^ Clinical Genetics, Guy's & St. Thomas' NHS Foundation Trust London Lambeth, Greater London UK; ^7^ Clinical Genetics, St. Michael's Hospital Bristol County of Bristol UK

**Keywords:** arts‐based methodologies, genetics, health research, patient and public involvement and engagement, reproductive health

## Abstract

**Introduction:**

When a couple has a child with a genetic condition, it raises questions when considering future pregnancies. It has typically been considered an easier genetic test result to receive when the condition is de novo (new) in origin, because neither parent is understood to have passed on the genetic change, except in rare cases. However, new scientific research indicates de novo changes are more likely to originate from a single sperm—a risk that increases as fathers age. Research on a personalised recurrence risk assessment strategy is underway (the Impact of PREGCARE study—‘iPREGCARE’). Couples may find this new information on de novo risks from fathers challenging without wider public awareness. This is a reflective case study of using co‐produced theatre to address this need.

**Methods:**

A range of participants (healthcare practitioners, those with relevant lived experience and the public) were creatively connected through scriptwriting to democratise knowledge production and research communication. Public and Patient Inclusion and Engagement (PPIE) participants were then asked to watch a staged reading of the playscript, to write letters to the characters, and to participate in a post‐performance workshop.

**Results:**

The staged reading and workshop were attended by 21 people; and over 336 people have viewed the publicly available recording to date. Qualitative analysis of the letters and workshop transcript indicate a major theme of informational discomfort. Two further themes, time pressures and support, focus on what the participants thought was needed but is often compromised or lacking.

**Conclusion:**

This approach created an opportunity to experiment with the benefits of theatre as a means to share the complicated dilemma couples may face due to new genomic information. Theatre, combined with the opportunity for the audience to write letters to the couple, provided an interactive means of communicating novel research on new reproductive genetic risk information to the public in a tangible and relatable form.

**Patient or Public Contribution:**

Six co‐production panel members, two additional public contributors and two actors worked with the researchers to develop and deliver the project. A series of meetings and an online collaboration space were used to refine the key messages, approve the script and consider evaluation. The co‐production panel reviewed and suggested edits to the draft journal manuscript.

## 1 Introduction

### 1.1 Background to the Research

Genetic diagnoses for severe paediatric disorders caused by new genetic variants, also referred to as de novo, affect approximately 3500 births in the UK each year [1]. This number is greater than the total combined cases of trisomies 13 (Patau Syndrome), 18 (Edwards Syndrome) and 21 (Down Syndrome), for which there is general public awareness and genetic screening is routinely available. The focus of our underlying research study (the Impact of PREGCARE study – ‘iPREGCARE') relates to understanding how parents of a child with a de novo‐related genetic condition perceive reproductive risks and their opinions on the potential benefits and drawbacks of population average risk versus personalised risk assessments.

The chance of recurrence of the de novo variant is usually given in the results appointment with the paediatrician, another specialist consultant (such as neurologist) or a consultant geneticist. Further information provision and psychosocial support may be given by a genetic counsellor, although not all couples receive this support. As the genetic conditions caused by pathogenic de novo variants are often rare or very rare, the condition may be unfamiliar to the specialist and further information may take the form of an information leaflet produced by a charity such as Unique (rarechromo.org).

The couple receive a substantial amount of unfamiliar, technically complex and potentially emotionally weighted information within a relatively short consultation. Currently, healthcare professionals inform parents that there is a 1%–2% chance of the de novo variant recurring in a future pregnancy. However, this population average does not reflect the true risk to the individual couple because the population average does not distinguish the majority of couples who have a negligible risk from those couples with a significantly higher recurrence risk. This happens when the de novo variant is present in multiple gonadal cells (sperm or eggs) of one parent (mosaicism).

At this stage, the nuance around the recurrence risk of the de novo variant may not be the couple's primary focus, increasing the likelihood of later uncertainty and confusion. Interpretation is further complicated by the fact that generic percentage‐based generic risks are not uniformly interpreted: individuals make sense of probabilistic information depending on their personal circumstances and prior experiences [2, 3]. Additionally, partners within the same couple may interpret the risk differently, adding another layer of difficulty when making decisions together about future pregnancies. As a result, some may avoid further pregnancy altogether, while others may seek additional testing such as Chorionic Villus Sampling (CVS) which itself presents an additional decisional burden, particularly because a positive result may lead to pregnancy termination [4].

To address this, a series of studies has been undertaken to develop a personalised pre‐conception risk assessment for couples and also to document the perspectives of both practitioners and families. The current study, iPREGCARE, examines couples' experiences of receiving de novo reproductive risk information [NIHR207257] [5]. Seeking broader engagement with this emerging area of genomics and to involve a range of stakeholders and public audiences in this research, we developed a complementary theatre‐based project. It is this co‐produced theatre initiative that is the focus of this reflective case study.

### 1.2 Arts‐Based Public Involvement and Engagement

Impactful research relies, at least in part, on meaningful patient and public involvement and effective dissemination of research findings to the audiences most likely to benefit or be able to take action. While traditional approaches often fail to engage non‐academic audiences [6], creative methods have been established as effective at doing so by supporting self‐reflection and longer‐lasting learning experiences [6, 7, 8, 9, 10, 11].

The aim of our theatre‐based project was to place the ethical and scientific issues surrounding a de novo genetic diagnosis in a child, and the associated uncertainties around recurrence risk in future pregnancies, in a context that was accessible and engaging for the public. This paper is a reflective case study of our use of applied theatre, drawing on both a novel clinically applied research project and the insight of a panel of co‐producers.

Genetic risk is often unfamiliar information for recipients and the public. Perspectives on risks may differ to those of the health professionals' providing the risks [2, 3]. For example, low risk results, such as those associated with de novo genetic changes, are not always perceived as reassuring [12, 13]. Additionally, de novo genetic risk carries a message of uncertainty regarding the risk of recurrence for any specific couple, not only because they are given a population average risk, but because they are told that there is a small possibility of either parent being mosaic for the genetic change—this is when it is in some of the mother's eggs or some of the father's sperm. They do not know whether they are in this small higher risk group or not. It is well documented that individuals will rely on their intuitive risk perception and risk acceptability when dealing with uncertainty of this nature [14, 15]. Their perception is driven less by objective probability and more by their interpretation of their own subjective risk—influenced by life experience, personality and beliefs—and their own feelings about the acceptability of the risk being considered [2, 3, 16, 17]. For this reason, theatre was utilised as a PPIE tool for exploring and communicating this personal and emotional complexity in how risk information is received.

Theatre has been used as a methodological tool in research on personal experience and sensitive topics [6, 18], and in works on health and health policy [11, 19, 20, 21, 22, 23]. Its application to communication or dissemination of research on genetic health and genetic testing is more limited, although several groups have used performance‐based methods to explore public understanding and engagement [10, 24, 25, 26]. For example, although explicitly empathic engagement is not a common approach in genetics, Nisker et al. integrated scriptwriting and theatre for a project on the fear of breast cancer, to educate and convey scientific, clinical, and psychosocial issues of adult predictive genetic testing, and to foster empathy for individuals navigating these experiences [23]. Mason et al. more recently worked with a youth theatre group and a PPIE group to develop two short plays about genetic risk information that could be captured by Polygenetic Risk Scores (PRS) [10]. These plays were shared in a workshop with patients and members of the public to facilitate discussions about PRS and their perceived benefits, concerns and emotional reactions. More broadly, use of theatre for empathetic engagement has also been investigated as a potential educational tool to teach doctors clinical empathy, the skill of recognising the emotional status of patients and family members and responding [27, 28, 29].

### 1.3 The ‘Making It Personal’ Project

The co‐produced theatre project was initiated and co‐ordinated by the iPREGCARE research project researcher (AK) and a drama research fellow and theatre maker (MJ). ‘Making it Personal’ was conceived as a theatre‐based initiative to involve and engage individuals with lived experience (e.g. people with experience of making family planning decisions based on genetic information), healthcare practitioners and the wider public with the issues surrounding de novo reproductive risk information.

The play was performed as a staged reading at an in‐person event in May 2025 and a recording of this was later made available online. The staged reading was followed by a workshop discussion and actor‐led improvisation session, in which attendees proposed additional scenarios for the actors to explore. The live staged reading and workshop was attended by 21 people, with a further 336 views of the recording (as of 1 July 2026).

### 1.4 Objectives

This project aimed to disseminate research on de novo recurrence risks and to illustrate the challenges couples can face when interpreting, discussing and applying this risk information in their family planning. Because reproductive risk is inherently sensitive and can evoke feelings of blame and guilt, an approach capable of eliciting empathic engagement was required. The primary objective was to engage the public with these issues and to create a reusable resource (a playscript and recording) [30]. A secondary objective was to build skills, capacity and experience in using creative PPIE and co‐production methods.

## 2 Materials and Methods

### 2.1 Co‐Production Panel

#### 2.1.1 Developing the Script

Potential co‐producers were approached during the development of the funding application (October 2024). Panel members were drawn from different stakeholder groups in order to represent a range of life experience and professional perspectives during the script development process: a clinical geneticist, a genetic counsellor, a professor of human genetics, a parent of a child with a genetic condition, an individual living with a genetic condition, a sibling of an affected person, and a member of the public interested in communications around health issues. Several members also held roles (paid or voluntary) in rare disease patient support organisations, providing insight beyond their own family circumstances. Most co‐production panel members identify as female and one as male; two co‐production panel members identify as British Indian, and the remainder as White British. All co‐producers are co‐authors of this reflective case study. Although unable to join the co‐production panel due to time constraints, two members of the iPREGCARE research study Public Advisory Group (PAG) also contributed through an individual hour‐long discussion with the principal investigator (AK).

The researchers and co‐production panel collaborated over several months (January ‐ April 2025), to translate ideas and experience into key messages and scenarios that would shape the playscript. This was carried out through online meetings facilitated by AK or MJ, a shared online Miro storyboard and later a jointly edited online draft (GoogleDoc). In this way, we created ‘blended’ stories' [19], which used multiple individuals' insight and reflections to represent issues pertinent to individuals, parents, families, healthcare practitioners, and researchers involved in the iPREGCARE study. AK and MJ shared relevant publications, as did other members of the panel. AK offered timelines for action and added ideas and feedback from meeting ideas to the Miro storyboard, which acted as editable ‘sandbox’ space for the consolidation of contributions into characters and scenarios.

To steer this collection of ideas towards a blended story, early sections of the script were drafted separately by the project lead and iPREGCARE study researcher AK, and panel member MP‐N (who is not a healthcare researcher or healthcare practitioner). These drafts were shared with the rest of the panel for discussion. This was then collated and finessed into a cogent whole by MJ, one of the co‐project leads, who is experienced in theatre scriptwriting and theatre making, before being shared for feedback again. The eventual blended story was a day in the life of a couple, Jess and Sam, who have just received a de novo diagnosis for their first child, Evie, and their conversations on whether or not to have a second child. As well as being part of the writing process, the panel members contributed ideas for visuals and props throughout, including the use of simple animation to depict the couples' social media interactions. The script was then rehearsed with professional actors Henry Charnock and Laura Hopwood. They also provided valuable feedback as they became the characters, drawing on their own experiences as either parents or family members, which significantly informed the interpretations of the characters and the final performance.

#### 2.1.2 Rehearsing the Script

The staged reading included the choreographed use of sounds and visuals. Background environment sounds and music were used to enhance audience immersion in the fictional couple's experience, drawing on evidence that natural sounds and music can impact emotions and attention, memory, problem‐solving, decision‐making, and creativity [31]. In particular, the inclusion of a sound effect of a child crying acted as a sensory reminder of the couple's lived reality of caring for a child whilst navigating this challenging emotional terrain. Visual cues focussed on the couple's use of their smart phones, searching for genetic information [32], exchanging messages with friends and family and receiving notifications from a parent group chat, highlighting the noisiness of living in a digital society, which can both support and overwhelm.

### 2.2 Staging the Play and Workshop

The staged reading of the playscript and accompanying workshop were advertised through the local media, circulated via a university PPIE mailing list, and by rare condition charities. Participation involved a two‐stage process: expressions of interests from those aged 18 or above were initially registered via an online form, after which an email was sent with event details, joining instructions, information about £50 reimbursement and request to confirm attendance. In this sense, the participants were a self‐selecting sample, although to encourage thoughtful engagement, at the expression of interest stage they were required to include a sentence about why they wanted to participate.

In a break between the performance and workshop, audience members were given the opportunity to write letters to the characters. The hour‐long post‐performance workshop was designed to facilitate post‐performance dialogue, capture immediate responses to the performance and gather feedback relevant to both our PPIE practice and the iPREGCARE study. The discussion was semi‐structured, led by AK, beginning with a Q&A with the actors on their experience of taking part, and then broadening to a wider group discussion, using prompts to encourage conversation. This was followed by an improvisation session, where audience members offered prompts for the actors to explore alternative ways that the fictional couple might have navigated their situation, or further conversations they may have had. The aim of this was to transform the participants ‘from spectators into active protagonists’ [33].

### 2.3 Analysis

We wanted to explore different perspectives and understandings of the material presented in the play. Thematic analysis is appropriate for an exploratory analysis of this nature that is not driven by existing theory [34]. The data consisted of the participants' letters to the characters in the play and the transcript of the post‐performance workshop discussion. The letters were transcribed by AK into one document. This document and the workshop transcript were then managed using NVivo software. The letters were also photographed so that they could also be read in their original format. We utilised the ‘OSOP’ (one sheet of paper) analytic method [35]. AK and MJ initially worked independently to close read all the data before each undertook inductive coding by reading and re‐reading the body of data as a whole and created their own summary view. These summaries and codes were then discussed together and refined to create a thematic plan [35]. The themes were shared with the project team for comments and further refinement.

### 2.4 Evaluation

As this is a reflective case study, we are also reporting on our tools for evaluating this PPIE project. Although evaluation of arts‐based approaches can be difficult in practice [7, 25], an evaluation plan for PPIE is considered good practice and was designed to capture both the impact of using theatre in raising awareness of the potential issues for couples in receiving de novo recurrence risk information, and also the co‐production process. In this way, evaluation was not designed solely to capture project metrics such as audience numbers or the amount of feedback. The project team kept observational notes throughout, and after the event participants and actors and co‐production panel members were requested to complete separate post‐event surveys delivered via Mentimeter. Eleven participant responses and six co‐production panel responses were returned.

### 2.5 Responsible Engagement

The iPREGCARE study obtained HRA and NHS REC approval [25/PR/0105]. The ‘Making it Personal’ PPIE project did not require separate ethical approval, however we followed responsible engagement practice as outlined in The Responsible Knowledge Exchange, Engagement and Impact (RKEEI) Framework: https://www.socsci.ox.ac.uk/rkeei Our actions our outlined in Table 1. The RKEEI framework encourages researchers to think about how research impacts those implicated. Given the sensitive and ethically challenging nature of the topic in question, this framework was considered particularly important.

**Table 1:** Responsible Knowledge Exchange, Engagement and Impact (RKEEI) actions.

RKEEI principle	What we did
1. Integrity & Ethics	Research Integrity training completed by project leads.
	Regular reflexive review by co‐leads.
	Miro board and Google Docs managed by invitation only.
	Workshop discussion: transcribed by University of Oxford
	approved partner with data management standards;
	participants de‐identified for privacy.
	Consent for recording obtained from participants.
2. Equity, Inclusion, Diversity and Belonging	Co‐production panel: demographically diverse and representing a range of stakeholders.
	The actors' reflections after performing the characters in the play provided a key element in the workshop discussion.
3. Reciprocity & Sustainability	Working remotely with digital tools.
	Reimbursement payment available to co‐production panel and participants at live event.
	Respecting the Equity payment rates for professional actors, and also providing their travel and subsistence expenses.
	Filming of staged reading to facilitate further sharing with wider audience.
	Using a venue with a Sustainability Action Plan.
4. Contextual Sensitivity and Cultural Respect	Working closely with people with lived experience.
	Communicating pre‐event and during event that we were not offering a ‘right answer’ but rather a springboard for discussion.
	Providing opportunity to leave open feedback in post‐event survey.
5. Sharing and Openness	Having a co‐production stage, working closely with those with lived experience.
	Online collaboration tools provided full transparency as ideas developed.
	Circulation of draft script before finalisation.
	Promoting event across multiple channels, including the RDM PPIE mailing list, various relevant charities, social media and a local newspaper (Oxford Mail).

In addition to applying the RKEEI Framework in our planning and delivery, the project team were mindful of the importance of strategies to mitigate potential harms to participants due to the sensitive nature of the content. A short “descriptor” of the content of the play was included on the website hosting the booking link for the staged reading. The audience were also reminded of this at the start of the live event and reminded that they could leave at any point and that a team member was available in the lobby if they wished to speak to someone. They were also advised that a 15‐min break would follow the performance, and this allowed audience members to leave less conspicuously if they so wished. Implied choice to participate was accepted if the audience member remained in the theatre.

Mitigating harms for the researchers, co‐production panel and actors consisted of tools that allowed for flexible working such as the Miro storyboard and flexible rehearsal times to accommodate childcare and other work commitments. The team included two senior academics experienced in mentoring and qualitative research management. This project was co‐led by AK and MJ and this created a buddy system for reflecting on issues and tasks. As the topic matter was new to MJ, AK supported with specific discussions to provide a grounding in the subject matter. Throughout, AK and MJ checked‐in with individual co‐production panel members and both actors to identify needs and mitigate any potential practical or emotional challenges in working on a sensitive subject matter throughout the process.

The intention at the outset was also to create resources that could be used again. The staged reading was filmed and has subsequently been made available to the public and shared with members of Unique, a rare disorder patient support group. At the time of writing (May 2026) it has been viewed 336 times. The playscript has also been published and deposited in the University's digital archive and made freely available to the public. It has been viewed 146 times and downloaded 49 times at the time of writing (1 July 2026).

## 3 Results

The co‐production approach to scriptwriting resulted in a playscript with key messages and scenarios drawn from patient and practitioner experience and insight. Combined with the skill of professional actors performing a staged reading, this playscript transformed the focus of the iPREGCARE research study into a format that was accessible to the public and clearly resonated with all involved:

It was a privilege to experience. I'm in awe of the way that the researchers, director, actors and support staff worked together to create something that was truly engaging.

Audience member

There was something very poetic for me as I sat and watched it with my husband and 4‐week‐old baby having been through the journey that Jess and Sam so beautifully acted out very recently. At the end, my husband turned and said that it was spot on and it felt like watching our story play out on stage and it captured the complexity of the situation.

Co‐producer

### 3.1 Staged Reading and Workshop

Actors Henry Chadwick and Laura Hopwood portrayed the characters of Sam and Jess. The audience for the staged reading and workshop was made up of healthcare practitioners, researchers, and members of the public who had responded to an advertisement distributed via several channels: local press (The Oxford Mail); patient support organisations (Unique and Genetic Alliance); various University of Oxford mailing lists for public involvement including the Centre for Personalised Medicine; and the social media channels of the organisers. 28 people registered; 21 attended on the day and took part in all activities (watching the performance, writing a letter, the workshop discussion). Responses to the anticipatory word cloud (*n* = 15) indicated that ‘understanding’ was primarily what participants were hoping to gain from their participation. They were expecting the PPIE to be ‘thought‐provoking’, ‘engaging’ and ‘immersive’, but there was also an element of uncertainty met with ‘intrigue’ and feeling ‘curious’ about an approach they found to be ‘novel’.

Immediately after watching the performance and before the workshop discussion, audience members were given time to each write a short letter to the characters Jess and Sam (see Table 2). Twenty completed the task and one misunderstood the task and wrote to thank the organisers for the event, saying it was ‘true to life’. Most letters were directed to both Jess and Sam, with only one participant choosing to send separate messages to each.

**Table 2:** Participants' letters to Jess and Sam.

*L1: Goodness what a lot of information to have to absorb in a 40‐minute appointment. No wonder heads were spinning. The feeling of isolation when people try to support was so sad ‐ I hope your mum's figure out how to be there. You were wonderfully there for each other though*.	*L2: You are faced with so much uncertainty. Talk openly and you will make the decision that feels right for you both*.
*L3: To Jess: Relax ‐ you have time to consider. You've dealt with so much, you'll think through this one*.	*L4: I think you ought to take a spectral view for yourself, your partner, and everybody involved. Consider time for self‐reflection ‐ the ripples of your own actions. Think about the notion happening to you. What would you want for yourself? Rid yourself of solipsistic nature. Develop a wider understanding of ‘needs’. Don't be afraid to ask questions, and don't perpetuate and expect the worst outcomes. Stress is the main killer. Allow time to breathe. It's okay if you find yourself digging a hole for yourself, as long as you remember that you can refill it. La essence, stop projecting and focusing on the issues talk about the solutions*.
*L3: To Sam: Reconsider the test. It's not about being guilty of something. It's about working out risks. Accept what science can offer!*	
*L5: How could your friends and family have better supported you through this journey of (1) having a child with additional needs (2) understanding the genetic result? Also, do you think the appointment with the genetic counsellor could have been done differently to leave you less confused?*	*L6: Just tell everyone that the problem has nothing to do with your family genetics and don't assess rare events as common unlikely to recur*
*L7: Your strength as a couple and sensitivity towards each other is inspiring and will see you through*.	*L8: Life is a funny business, but it does go on. There are no right or wrong answers, but not making a decision will always be a roadblock on a journey. And too much information is not always helpful. Best of luck. Life is a strange journey*.
*L9: You seem like such a strong couple. How can you take the ‘support’ of friends + family without being put off by negativity?*	*L10: Hang on in there, what you are going through must be so incredibly difficult. Genetic, de novo or otherwise, is out of our control. It's not your fault. If it feels like too much it's okay for you not to choose to have more children, but if you do my best wishes for an easy and complications free pregnancy and baby*
*L11: Such a difficult decision but take your time ‐ Try to get more counselling to help understanding more about what the research tests are. Can you ask your parents to support you in different ways ‐ listening more ‐ whatever can reduce the stress*	*L12: Firstly, my heart goes out to you. Also know you are not alone ‐ all parents go through some elements of assessing risk and uncertainty. I think if I was in your situation I would try for another child based on your current risks. More knowledge may not help*.
*L13: Would talking to those in similar situations help ‐ risks are difficult to assess alone but knowledge helps. Good luck*	*L14: Good luck whatever you decide to do*.
*L15: Being honest and respecting each other will take you far…even the wise cannot see all ends. You are doing great!*	*L16: What about if it turned out that everything would be absolutely fine? No need to torture to each other…*
*L17: Knowledge is power*.	*L18: It looks like counselling is a good option after such news. And information (reliable)/website would help answer some questions. It's always better best to leave the big decisions for later because of the shock effect*.
*L19: You shouldn't have so much worry, or stress. Ultimately it is not about the test results, it is about the available on quality of support that you can give*.	*L20: Give yourself time to process it and be gentle on yourself and seek someone to talk to for information/support/counselling*.

Analysis of the letters (L1–L20) and the transcript of the post‐performance workshop discussion, which including the participants, the actors and some members of the co‐production panel (F: female and M: male participant), indicated a major theme of informational discomfort. Two further themes, time pressures and support, focused on what the participants thought was needed but is often compromised or lacking.

#### 3.1.1 Theme 1: Informational Discomfort

Informational discomfort encompasses a number of sub‐themes. Firstly, information overload, which represents the times participants wrote or spoke about the difficulty of processing, absorbing and remembering the detail of new and unfamiliar information on the genetic causality of a child's health condition and the risks to future pregnancies:

Goodness what a lot of information to have to absorb in a 40‐minute appointment. No wonder heads were spinning.

[L1]

I feel like you could see them struggling to add de novo into their vocabulary and things like that and even if you can say the word, it's like it doesn't seem like, it definitely didn't seem like sounds 100% sure of what it means

[Actor who played Sam]

Second, informational discomfort related to sense‐making and the struggle to process and use imperfect information, information that leaves uncertainty, and the tussle between rational and emotional responses (ii):

You are faced with so much uncertainty.

[L2]

I wanted to comment on the 1% or 2% chance of it happening again, but I think for people when they have a child who already has a genetic mutation, for them it's 100%. Right? They've already lost the lottery to a certain extent, so their reality is going to be so skewed.

[Female workshop participant F2]

Thirdly, knowledge, knowing and participants' thoughts on the limits of what is true, real or knowable, which related to epistemic anxiety, indicating a polarity in their views regarding how much information is enough information. Some people saw additional information from personalised recurrence risk testing as empowering the couple's decision‐making:

…knowledge is power.

[L17]

Reconsider the test. It's not about being guilty of something. It's about working out risks. Accept what science can offer!

[L3]

Whilst others saw more information as potentially complicating the couple's decision‐making and anxiety:

…even the wise cannot see all ends.

[L15]

too much information is not always helpful.

[L8]

Within this sub‐theme of epistemic anxiety, there was also a comment on trust and information sources:

Just a word of caution. I think the challenges we have these days is algorithms and AI…stuff will be thrown at you and you end up being taken down a completely different path to what you figured to be going down…and then it looks like it's reality when it's not.

[Male workshop participant M4]

#### 3.1.2 Theme 2: Taking Time

The theme of time reflects the many different ways in which time was mentioned in the letters or the workshop discussion. This theme largely relates to the emphasis participants placed on how important it was for couples receiving genetic recurrence risk information to take time and not rush their decisions. In particular, many letter writers reinforced the importance of taking time to process the information and make a decision about testing or a future pregnancy that was right for the couple:

Give yourself time to process it and be gentle on yourself…

[L20]

Allow time to breathe.

[L2]

However, the participants were also sensitive to the time pressures of fertility and the biological clock that couples may also be influenced by:

…one thought that came to me was the time pressure you might feel under. So, you've already had a baby…most people are starting their families in their early 30 s now, you've waited two years for this appointment…you might be feeling those biological pressures of well if we're going to have another baby, it took us 18 months to fall pregnancy with Evie, how long is it going to take?

[Female workshop participant F5]

There are no right or wrong answers, but not making a decision will always be a roadblock on a journey.

[L8]

#### 3.1.3 Theme 3: Support Needs

Support was another key theme we identified in our data. Many letters included on the importance of self‐reflection and listening to each other and to see their strength as a couple:

Your strength as a couple and sensitivity towards each other is inspiring and will see you through.

[L7]

Being honest and respecting each other will take you far…

[L15]

However, the workshop discussion also recognised that this isn't possible for all couples and that they may need more psychosocial support:

Having another child after having a child with a genetic condition will probably or could possibly have a very different ending [to the play] where the relationship breaks down and maybe that's something that needs to be explored and actually calls for counselling that's much further than just the genetic counselling and the explanation of what's going on.

[Female co‐production panel member]

The letters and workshop discussion recognised not only the issues for the couple in not receiving the support they need but also the challenge for family and friends in understanding the diagnosis and risk and knowing what to say or do to be helpful and the isolation that can result:

It's isolated because no one gets it.

[Actor who played Jess]

The feeling of isolation when people try to support was so sad ‐ I hope your mum's figure out how to be there.

[L1]

As this one participant alluded, if it is burdensome to share, there may be a temptation to not tell family and friends:

Just tell everyone that the problem has nothing to do with your family genetics.

[L6]

However, there was a compassionate view towards friends and family and a recognition that it is hard for them to know what to say and that more support for them would be helpful:

Well, as a person how do you know what you're doing right in terms of yes, you want to be interested and supportive but also you don't want to pry and be a little too invasive? How do you balance that out?

[Female workshop participant F2]

Maybe there needs to be an information pack for family, […] people don't really know how to talk to you and either don't talk to you at all or say the wrong thing…

[Female co‐production panel member]

And that connecting with others with shared experience might be another source of invaluable support.

### 3.2 Post‐Performance Evaluation Survey

Eighty‐two percent of those who responded to the post‐performance survey (*n* = 11; 52%) strongly agreed or agreed that they had gained insight, a new perspective, or learned something new. The majority also strongly agreed that ‘Making it Personal’ was produced and presented to a high standard (Strongly agree 9/Agree 1) and that the team had challenged themselves with this work (Strongly agree 8/Agree 2). Participants were able to leave feedback, and this was uniformly positive (see Table 3). Their comments reflected how powerful and different it felt to learn about issues raised through research through theatre and many remarked on how emotionally resonant and thought‐provoking the experience was, for example: *“It really brought the ideas to life in a way that allowed the complex layers to come through with nuance and emotional impact.”* Crucially, co‐produced theatre offered *“…an immersive route to exploring difficult ethical questions”*.

**Table 3:** Participants' qualitative responses from the post‐performance survey.

Responses to ‘Theatre is helpful for engaging the public with scientific research and its social implications':	
*It's easily understood*.	*Creative expression of research findings helps to put them in a human context if the subject matter is appropriate*.
*I think it brings to life the issues people can face and helps to make complex topics more accessible to the public*.	*Any novel way of dealing with such sensitive and personal issues can only be applauded. I thought it triggered a new understanding in my heart*.
*Theatre can reach a range of people that would not usually engage with similar questions as well as provide an immersive route 2 exploring difficult ethical questions*.	*The performance is a very creative way to disseminate research findings and to provoke discussion from participants. The element of surprise in this medium invites genuine interaction from the audience*.
*It forces you to consider a new perspective*.	*It can bring things to life in an interesting way*.
*The actor's comments* [in the workshop] *added much to the impact of the scripted stuff*.	*It really brought the ideas to life in a way that allowed the complex layers to come through with nuanced and emotional impact*.
Responses left under ‘General comments’:	
*Excellent afternoon with stimulating content*.	*It was a wonderful show really immersive despite the lack of settings stroke/props*.
*I really felt humble. There must have been a lot of work and collaboration. I don't have much experience in acting but making it as real as possible must have been a challenge*.	*I acknowledge funding is not limitless but would encourage committees to support future applications when these are presented convincingly*.
*It was a privilege to experience. I'm in awe of the way that the researchers, director, actors and support staff worked together to create something that was truly engaging*.	*A minor admin point ‐ more clarity on the length of session would help vague people like me*.
Response to ‘Have you experienced theatre being used for engagement with research before?’	
*Yes, as PPI at a clinical psychology conference. A play was put on about a psychological scenario and then we as the audience were able to discuss the scenario and suggest alternatives be acted out*.	*At a different conference, so a play acted out by theatre group with learning disabilities, re certain social issues and assumptions re their ability to raise children*.
*This is my first time. I was interested to witness how it provoked great reflection from the audience*.	*No, however it's much more effective than cartoons*

In the context of this small, self‐selecting sample of participants, using creative PPIE succeeded in illustrating the potential sensitivity of risk information and the discomfort and possible conflict that a couple can experience. Respondents to the evaluation survey told us that they had mostly not experienced theatre in a PPIE context before and were unanimous that theatre is helpful for engaging the public with scientific research and its social implications (Strongly agree 8/Agree 2). The majority of those who responded to the feedback survey agreed that the event sparked curiosity and conversation with the people who attended (Strongly agree 7/Agree 3/Strongly disagree 1). Although one person did not agree, they did not leave an optional comment and so we do not know why their perception differed to the others. However, it is important to recognise that theatre and group discussions may not be everyone's preferred method for engaging with research.

#### 3.2.1 Demographics

Those who attended the performance and workshop were healthcare practitioners, researchers and members of the public. Responses to the post‐event demographic survey were very limited (*n* = 8) but indicated a broad age range amongst participants (25–65 and over). Respondents were largely drawn from Oxford or London, were English language speakers at home and educated to degree level. Seventy‐five percent of respondents to the demographic survey identified as female.

### 3.3 Co‐Production Feedback

The co‐production panel were also invited to provide feedback on their experience. Their responses via survey and via email indicated that the project had provided them with an opportunity to reflect professionally and personally. They reported feeling they had improved their ability to communicate complex genetic information in accessible and resonant ways. In the words of one panel member, being involved in the project has helped them to “*reflect on my own day to day work, to collaborate with other professionals and patients, and consider how to present complex information in a meaningful and accessible way*.” The process was new to most people involved and it was really meaningful for the co‐producers to *“see the characters come to life and our initial conversations and ideas grow into this magnificent, thoughtful and provoking script.”* The staged reading of the play and its recording was a powerful vehicle for capturing all the blended stories and insights that the panel had shared: *“I was blown away by the play”*; *“this was an excellent way to share such an emotive and complex issue”*; *“the end result was a fantastic mosaic woven from the unique perspectives each member shared…It covered so many themes and did so with such sensitivity and depth”.*

## 4 Discussion

Arts‐based approaches to public engagement can be used to create spaces where ambiguity and emotion can be shown as relevant to research and the public can be introduced to research topics they may otherwise not have encountered [7, 30]. This is important for supporting engagement with potentially sensitive subjects such as genetics, pregnancy and risk, which have social implications beyond the individual [11]. Participants in this project expressed that engaging with the focus of our research through theatre provided a more emotionally resonant understanding of these complex issues and helped bridge gaps between clinical information and lived experience. By particularising this complex information and challenges to a fictional couple, we managed to portray a story which was judged authentic and resonant. Using theatre to communicate the challenge for couples receiving this type of genetic information synthesised the rational and the emotional, the imaginative and the intuitive. Bringing an innovative dimension to problem solving through PPIE provides an illustration of how to better support couples and their families. For example, our participants identified the gap in support and wondered if online information packs to facilitate better supportive conversations could be created.

Empathic engagement is an approach not commonly taken in genetics, however there have been a handful of similar initiatives. For example, Nisker et al utilised scriptwriting with contributors to develop a piece of theatre to be used in 12 case studies on the fear of breast cancer [23]. More recently, Gorman and Farsides used ‘participatory‐writing' to understand how the diverse, multilayered everyday lives of families correspond with the emerging, rapidly changing and complex field of genomic medicine [36]. Whether script, poetry or letter writing, this process of creation can provide insight into personal and sensitive topics such as genetic risk because perception is driven less by objective probability and more by each individual's interpretation of subjective risk, influenced by personality, beliefs and the experiences life brings [2, 3, 16, 17].

Having a co‐production process was essential to this project because it democratises knowledge and blurs boundaries between knowledge users and generators [19]. In a recent systematic review of the use of creative PPIE in health research, Philips et al observed a strength to be the cultivation of a feeling of equality between academics and the public [11]. Not only does this expand intra‐ and extra‐ organisational capacity for knowledge mobilisation and research [37], it encourages and creates opportunities for active agency in improving understanding and care and communicates to the wider public that lived experience matters [19, 38].

For the researchers involved, this project expanded knowledge of effective engagement methods, enhancing our skills in using theatre as an engagement tool. Awareness of positionality was shared openly throughout the process. AK is a researcher and also a parent of a child with a de novo diagnosis. MJ is a researcher (non‐health related) and an experienced theatre maker. When offering their views, ideas and comments, other panel members also shared their own positionality, for example as a parent, an affected person, a health practitioner, or a member of the public. The co‐development of the script took place over a series of online meetings and via an online collaborative tool called Miro [miro.com], an approach designed to accommodate collaborators from various locations and with other commitments. The meetings were initially more successful at gathering ideas and feedback than the online board. However, the Miro board was useful for holding and organising those ideas and providing structure to the script development process, moving the team on from free‐form ideas to the development of various scenes in the script.

Research on the effectiveness of digital tools for remote collaboration and communication, or on the soft skills required to use them, is limited [39], with studies focused on the benefits and challenges of remote synchronous meetings and not other non‐synchronous online collaborative tools [40, 41]. Whilst in our case unfamiliarity and working‐style preferences was primarily the issue regarding uptake of the digital collaboration platform, it would be important for future studies to consider challenges such as digital fatigue, the loss of observable social cues and the potential for digital inequalities when using this approach to collaboration [42].

### 4.1 Strengths and Limitations

One of the key strengths of our methodology is its novelty and the inclusion of a carefully selected co‐production panel that reflects an inclusive approach to script development. This PPIE project also addresses the challenge of communicating complex, emotionally laden genetic information, which is timely given evolving understandings of de novo genetic risk and the challenges of communicating uncertainty. It offers novel methodological insights in its use of letters to characters as a form of data collection and engagement.

Although we have applied qualitative approaches to consider and share our experience in the form of a reflective case study, we acknowledge that our findings are exploratory and based on a small sample of participants that were largely self‐selecting, and there will be potential bias. There are also contextual limitations. The characters of Sam and Jess are based in the UK and have access to care via a National Health Service (NHS). Although mixed race actors (White British female and British Indian male) had been secured at the time of grant writing, other commitments meant they were unavailable at the time of staging. Despite a colour‐blind casting call and interviews, the actors who ultimately played these roles were both White British. As the actors are an important element in representativeness and final script development, this will have created a bias. However, the script has been made open access to create opportunities for further adaption and development.

As was the case for other theatre‐based approaches [23, 25], a key limitation we faced was time. For the core team, it was challenging to balance this ambitious engagement project alongside research roles. Co‐producers also had limited time, and it was sometimes difficult to schedule mutually convenient meetings. In particular, scriptwriting can be a lengthy process. For example, Nisker et al. reports that their playscript on adult predictive genetic testing took a year to write [23]. The development and writing process for ‘Making it Personal’ was completed within 5 months, which was challenging.

A second impact of the time constraint was that the staged reading was held within the university infrastructure. The venue may have influenced the education level of the participants, which is an important consideration. The project team were cognizant that choice of the right venue is crucial in eliminating barriers to participation and initial efforts were made to secure space at a community theatre, but this was not available until after the grant spend deadline. Taking the live event into the local community might have attracted a more diverse audience. The budget did allow for professional recording of the staged reading however, and this is an open resource, along with the published play script.

As Philips et al.'s systematic review noted, matching the creative PPIE approach to the topic and the participants requires careful consideration [11]. Although the feedback for our initiative was extremely positive, one attendee of the live event did not feel that a theatre‐based approach was for them, and this is valuable feedback. It may be that creative approaches, which are less often utilised in genetic health research engagement, may not feel comfortable for everyone.

## 5 Conclusion

Although a small exploratory study, there are implications for practice when it comes to sharing genetic information, including recognition of the informational discomfort faced by couples provided with population average risks for their child's de novo genetic condition. Time is a limited resource for health practitioners and couples and yet it may be needed to work out what the information means for them as individuals and as a couple. The third theme of ‘support needs’ highlights there is likely value in providing a support guide for their friends and family, which could remove some of the explanatory burden from couples and provide some scaffolding for supportive conversations.

The implication for genetic research PPIE is that theatre offers a platform to combine abstract, unfamiliar and complex research with social and experiential realities. In particular, this reflective case study demonstrated theatre‐focussed PPIE is a useful methodology for exploring the ambiguity of genetic risk information and tackling the emotional and practical implications of uncertainty. Humanising probabilistic information through a dramatic narrative helps to make research on genetic risk more tangible, memorable, and relatable to life decisions for the public. Extending this reach with an active element such as physical letter writing, encourages depth of thought from the audience members, to organise their reflections in response to the story, and to connect what they have learned to their own responses to health, risk and family communication.

This reflective case study has potential implications for the practice of co‐production itself. Co‐production is at the heart of this methodology. It drew in diverse knowledge, engaged emotional responses through drama and storytelling, and created multiple opportunities for social interaction through the script development, scriptwriting and staging. Digital tools were used to reasonable effect in this study but alternative channels for idea sharing and decision‐making, particularly meetings, were essential. Building trust and relationships is vital and regular meetings, albeit online and not in person, were aided but not replaced by the availability of non‐synchronous digital tools.

Finally, impactful health experience research requires meaningful engagement, not only with those directly impacted by the focus of the research but also healthcare professionals and the public, especially in the area of genetics because a genetic diagnosis or risk is often not limited to one person in the family. Given adequate funding, time and planning, this case study demonstrates that PPIE using theatre is a worthwhile method of building understanding and generating ideas with the public—the nature of which may not be otherwise generated using traditional methods.

## Author Contributions

**Alison Kay:** conceptualisation, writing – original draft, writing – review and editing, funding acquisition, investigation, methodology, data curation, formal analysis, project administration. **Anne Goriely:** writing – review and editing, funding acquisition, investigation, methodology. **Alexa Gummow:** investigation, writing – review and editing. **Lisa Hinton:** funding acquisition, methodology, writing – review and editing, investigation. **Arti Patel:** investigation, writing – review and editing. **Marysia Price‐Nowak:** investigation, writing – review and editing. **Shwetha Ramachandrappa:** investigation, writing – review and editing. **Jonathon Wells:** investigation, writing – review and editing. **Francesca Wicks:** investigation, writing – review and editing. **Minna Jeffery:** methodology, conceptualisation, investigation, formal analysis, funding acquisition, project administration, writing – review and editing, data curation.

## Conflicts of Interest

The authors declare no conflicts of interest.

## Ethics Statement

The ‘Making it Personal’ project was designed as a Public and Patient Involvement and Engagement for an NIHR‐funded research project (iPREGCARE: NIHR207257; HRA 25/PR/0105). Formal ethical approval from a REC is not required for PPIE activities in the UK.

## Data Availability

The data that support the findings of this study are available on request from the corresponding author. The data are not publicly available due to privacy or ethical restrictions. The playscript is available as an open source document: Kay A, Jeffery M, Goriely A, Hinton L. Making it personal — play script. Centre for Personalised Medicine, University of Oxford. 2025. doi: 10.5287/ora‐o1vyx8k1n. Available here: https://ora.ox.ac.uk/objects/uuid:f15b81bc-55d0-4e08-8f9d-9f15fb6e8f8d. A staged reading of this playscript is available via the Centre for Personalised Medicine (CPM) website: https://cpm.ox.ac.uk/event/making-it-personal/. Evaluation and transcript data is available on request to the corresponding author.
